# Advances in electro- and sono-microreactors for chemical synthesis

**DOI:** 10.1039/c8ra03406k

**Published:** 2018-06-19

**Authors:** Tomas Hardwick, Nisar Ahmed

**Affiliations:** School of Chemistry, Cardiff University Main Building, Park Place Cardiff CF10 3AT UK AhmedN14@cardiff.ac.uk

## Abstract

The anatomy of electrochemical flow microreactors is important to safely perform chemical reactions in order to obtain pure and high yielding substances in a controlled and precise way that excludes the use of supporting electrolytes. Flow microreactors are advantageous in handling unstable intermediates compared to batch techniques and have efficient heat/mass transfer. Electrode nature (cathode and anode) and their available exposed surface area to the reaction mixture, parameters of the spacer, flow rate and direction greatly affects the efficiency of the electrochemical reactor. Solid formation during reactions may result in a blockage and consequently decrease the overall yield, thus limiting the use of microreactors in the field of electrosynthesis. This problem could certainly be overcome by application of ultrasound to break the solids for consistent flow. In this review, we discuss in detail the aforementioned issues, the advances in microreactor technology for chemical synthesis, with possible application of sonochemistry to deal with solid formations. Various examples of flow methods for electrosynthesis through microreactors have been explained in this review, which would definitely help to meet future demands for efficient synthesis and production of various pharmaceuticals and fine chemicals.

## Abstract 2

The advances in flow microreactor technology for chemical synthesis, with possible application of sonochemistry to deal with solid formations.

## Graphical abstract

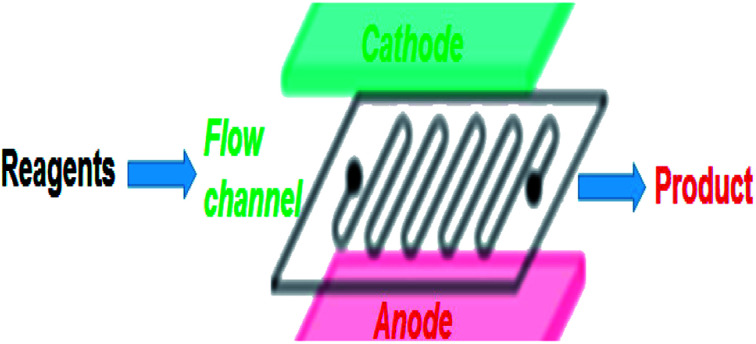

## 1. Introduction

Typically, chemists are used to performing chemical reactions in standardised glassware (what we refer to as “batch”) with various methods for heating/cooling and stirring. In addition, more advanced techniques have been employed, such as photochemistry, electrochemistry, ultrasound, and over the last two decades, microwave heating, and have all become a regular occurrence. By overlapping the fields of organic chemistry and chemical engineering, a new methodology known as “flow chemistry” has been developed where, in contrast to conventional batch chemistry, the chemical transformations are to take place inside tubes/pipes or micro-channels, termed continuous flow.^1^ Performing reactions in continuous flow rather than in batch has led to a variety of advances regarding safety and sustainability. The miniature chemical reaction device with micro-channel/s, known as microreactors, has been revolutionised in the field of organic chemistry. Initially, the field of microreactor technology for chemical synthesis was developed with notable contributions from scientists at GlaxoSmithKline (UK),^1*b*^ Massachusetts Institute of Technology (USA),^1*c*^ the Institut für Mikrotechnik Mainz (Germany)^1*d*^ and Imperial College London (UK),^1*e*^ amongst others. Many reactions can receive huge benefits by taking advantage of the physical properties of microreactors, such as enhanced mass- and heat transfer due to a large surface to volume ratio as well as regular flow setups leading to better yields with increased selectivity's. Stringent controls over thermal or concentration gradients within the microreactor allow new methods for efficient chemical transformations with high space-time yields. The traditional batch electrolytic methods suffer from a number of limitations such as heterogeneity of the electric field, thermal loss due to heating and mandatory use of supporting electrolytes. These elements either hinder electrosynthetic efficiency or make the separation process awkward. Therefore, electrochemical transformations seem to be particularly well suited to be performed in microreactors.^2–4^ The combination of electrosynthesis and microreactor technology can effectively overcome some of the difficulties in batch electrochemistry and achieve higher product selectivity's and purities, lower number of oxidation steps, fewer by-products and lower energy consumption. The potential for organic electrosynthesis in microreactors will considerably increase the application range of electrochemical synthetic methodology in both academia and industry.^5–8^ However, flow electrochemical microreactors have a particular problem with the handling of solids. Solid reagents as well as solids created in the reaction that agglomerate into larger particles may build up along the walls of a flow reactor, increasing the pressure drop and clogging the path of flow. These hurdles can in some cases significantly hinder a reactions capability to receive the full benefits from microreactors in organic reactions. It is, however, possible to break apart these agglomers with ultrasound irradiation which will open up the unique prospect of sonoelectrochemistry of particulates.^9^

Certainly, organic electrochemistry plays an important role for organic synthesis.^10,11^ However, there are some difficulties intrinsic to conducting electrode processes in organic media. For example, the conductivity of common organic solvents is low which usually results a higher cell voltage compared to that of aqueous systems and reactions at the electrode surfaces suffer from the problem of low mass transfer, therefore, resulting in a lower productivity compared with homogeneous systems. The design of suitable devices for electrolysis is, therefore, quite essential. Supporting electrolytes such as tetraalkylammonium salts also creates a problem of separation and, therefore, recycling after the electrolysis is required. These problems of conventional batch electrolysis might limit the applications of the organic electrochemistry, however, the application of microreactors serves as a solution to these problems.^12^ The small distance between the electrodes avoids a high ohmic drop. High electrode surface area-to-reactor volume ratio in electrochemical microreactors is also advantageous for mass transfer at the electrodes.

The anatomy of an electrochemical flow microreactor is very important for efficient electrochemical reactions. There are two main categories of microreactors, which are applicable for electrochemical synthesis; the first is an undivided microreactor, in which the working and counter electrodes are placed in the same flow channel, and the second is a divided microreactor, in which the working and counter electrodes are separated by a diaphragm (*e.g.* ion exchange membrane). The interference of substrates and products at the counter electrode in chemical reactions mainly decide the kind of microreactor. In the absence of such interferences, an undivided microreactor would be preferred. On the other hand, a divided microreactor can be used where the anodic and cathodic process do not interfere each other. The selection of conductive solvents, and the small distance between electrodes of microreactor (in the range of a few micrometres up to 1 mm) can allow chemical reactions to be performed without any supporting electrolytes. Thus, several types of electrochemical flow microreactors have been developed for their potential use in chemical synthesis.

Modern developments in flow chemistry, with respect to efficient mixing, parameter control and heat and mass transfer, have allowed miniaturisation of devices that have shown the potential to control experiments such as synthesis with unstable intermediates or very reactive reagents that would be difficult to conduct under batch conditions.^12*a*^ Although these flow reactors are generally smaller than batch reactors, optimised conditions enable more product to be produced per unit time than batch.^12*b*^ The use of this microreactor technology offers the chemist several benefits in comparison the conventional batch reactor:

Continuous operation – with the utilisation of automated pumps microreactors can be introduced to a continuous flow of reagents, thus removing typical workup time delays (beneficial in low temperature chemistry where reaction times are very short). In some cases, the decay of important intermediates are able to be avoided, therefore, offering better selectivities.^1^

Selectivity – microfluidic reactors offer an easier and more carful control over reaction conditions such that a greater selection over desired products is often possible, and hence there is a beneficial effect over yield and purity.

Efficiency – the increased surface area to volume ratio and small reactor dimensions results in a superior heat and mass transfer than a standard round-bottom flask, and mixing of reagents by diffusion is very quick. This is also true for an industrial scale where heat exchange between the reaction medium and reaction vessel is also highly efficient. Small distances between electrodes such that the two diffusion layers of the electrodes become “coupled”, allowing ions to be electrogenerated and play the role of the supporting electrolyte and a high heat transfer capacity that can be achieved with small diameter channels (important for chemical engineers).

Safety – health and safety are of paramount importance to any chemical environment. Microreactor properties enable them to safely handle, consume and generate hazardous materials: high surface area to volume ratios allow heat to be rapidly transported during exothermic reactions, and small dimensions allow toxic and explosive species to be dealt with.^12*c*^

Small quantities of reagents – obviously, where dangerous chemicals are involved, having to use less is a safety advantage. Reduced amounts of reagents are beneficial in terms of expenditure, particularly when expensive and/or minimal quantities of precious reagents are available. Microreactor flow systems, in most cases, consume less reagent than batch in order to obtain the same information (in the case of informatics).^12*b*,*d*^

Multiphase reactions – processes that involve solids, liquids, gases and supercritical fluids^12*e*^ can also receive benefits due to the increased surface area to volume ratios. Surface contact between the different phases inhibits undesired effects of mass transport,^12*f*^ resulting in greater mass and heat transfer. These reactions can have better yields and selectivity's of several gas–liquid and gas–liquid–solid reactions,^12*g*,*h*^ improved rates, controlled mixing of reagent streams and improved selectivity in studies concerning heterogeneous catalysis with packed-bed microreactors.^12*i*^

Rapid reactions – reactions in microfluidic reactors are rarely run for longer than required to reach the reaction endpoint, as they can be closely monitored to determine reaction completion. Referring to space-time yields (product formed per reactor volume and time), reaction rates have been reported to be higher than those performed in bulk reactors.^12*j*^

Green chemistry – the improvement in selectivity will reduce the amount of waste produced, and the efficient heat transfer will result in a smaller amount of energy consumed per unit temperature, leading to environmental benefits.^12*k*^

Recent attention – the field of flow chemistry (in microreactors) has now gained enough attention to make it a hot topic, increasing the number of research efforts focused on pushing this technology to the next level, enabling new equipment to become commercially available and from production and small-scale work performed in laboratories to scale-up to for industrial level applications, microreactors have evolved for purposes of reaction optimisation, to obtaining kinetic and mechanistic information,^4,12*l*^ as well as new (and hopefully improved) synthetic methods.

However, the specialised equipment required for microreactor flow systems also increases the difficulty of screening reaction conditions in conjunction to those commonly performed in batch.^12*k*^ Small distances between electrodes such that the two diffusion layers of the electrodes become “coupled”, allowing ions to be electrogenerated and play the role of the supporting electrolyte^4^ and a high heat transfer capacity that can be achieved with small diameter channels (important for chemical engineers) favours micro over mini flow reactors.^12*c*^ However, problems arise from the difficulty to screen reactions due to complex equipment setup, limited flow capacities, cleaning and dismantling, high pressure drops and a tendency to block.^12*c*,*k*^ Therefore, developing efficient optimisation conditions is particularly valuable for microreactors.^12*k*^ The development of inexpensive tubular microfluidic systems for reactions (microreactors), are often based on perfluorinated polymer or stainless steel tubes,^12*k*,*m*^ and has allowed a great deal of reaction types to be performed^12*n*–*t*^ by the organic chemistry community. In conjunction to this, the possibilities of flow have also been realised by the inorganic chemist who have begun to couple flow with heterogeneous catalysis. Scale-up of reactions today can be accomplished by a combination of increasing reactor dimensions with structures that preserve heat and mass transfer followed by multiplying reactors together. With a careful control of pressure, dropping a uniform distribution of fluids across multiple flow channels can be achieved.^12*u*–*w*^ Earlier studies of this simply used multiple reactors, however, this approach left highly challenging control and fluid flow distributions.

## 2. Progress in electrochemical flow microreactors and their applications

### 2.1. Reactors with parallel electrode configuration

Electrodes stacked in a parallel plate-to-plate fashion in which two electrode plates are separated by isolating spacers to produce a parallel flow channel has become a popular choice of micro-technology.^3,13,14^ This type of flow cell has now been reported for use in many laboratory electrosyntheses^15–23^ and commercial usage.^13,24–26^ All parallel plate cells can be run as divided or undivided cells. As elucidated below, the gap between electrodes should be as small as possible. When mesh electrodes are used in divided cells, the membrane surface and the electrodes may be in contact (with the feed to the reactants at the back of the mesh). This kind of set-up has been referred to as a “zero-gap” cell.^2^ A number of papers describe parallel plate flow cell designs suitable for laboratory electrosynthesis. Such cells have also been marketed by a number of companies. This can be attributed to several advantages of this configuration, as outlined below:

- A very small gap between the electrodes is easily achieved which may permit an electrochemical reaction without the need for an additional electrolyte, as well as thin concentration boundary layers, resulting in enhanced mass transfer rates between electrodes.^2^

- Recycling the flow can be avoided *via* a continuous process by operating in a single pass high-conversion mode.^2^

- Fabrication using plane plates allows easy replacement and therefore, can be made of almost any electrode material.^3^

- High specific area with all points on both the electrode surfaces are equivalent with respect to each other.^3^

- Uniform flow rate within the inter-electrode gap (uniform cell current and potential distributions); which should be as rapid as possible to give maximised mass transfer rates and hence maximum conversion rates.^3^

- Introduction of a separator without drastically changing the cell structure.

- Coupling multiple cells in parallel and/or altering plate areas allow simple scaling up.

**Fig. 1:**
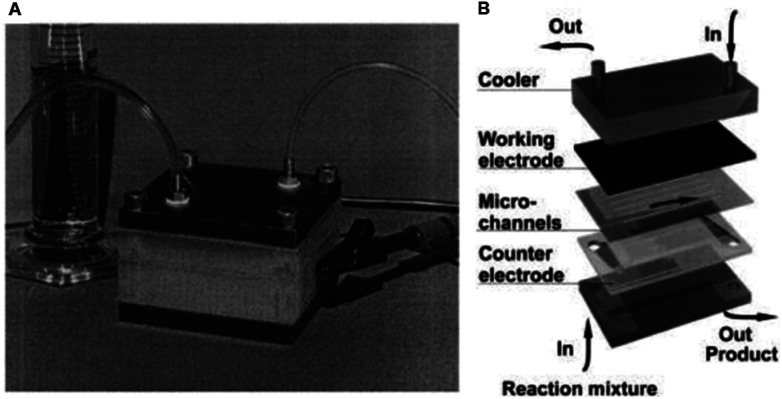
(A) Monochromatic photograph of the microreactor for electrochemical organic syntheses as developed by Löwe and Ehrfeld, and (B) its schematic assembly. Reproduced with permission from ref. 27, Elsevier.

Such a plate-to-plate configuration was fabricated by Löwe and Ehrfeld^27^ in 1999 whom applied this concept of microstructuring techniques for thin layer technology for the oxidation of 4-methoxytoluene to 4-methoxybenzaldehyde. The reactor geometry saw the two electrodes separated using polyimide foil (75 μm thick) with an additional multichannel built into the foil (Fig. 1). Using a glassy carbon anode, with 0.1 mol L^−1^ KF supporting electrolyte, quantitative conversion during the anodic oxidation of methanol was achieved, and with respect to the formation of the product, efficiencies that exceeded 98% were realised. It is also possible to perform the electrosynthetic reaction in continuous flow mode, enabling organic mass production processes to be introduced to this system. Due to the small electrode gap, the oxidation could be performed smoothly without the addition an electrolyte, although with a reduced conversion in comparison to the reaction with an electrolyte.

**Fig. 2:**
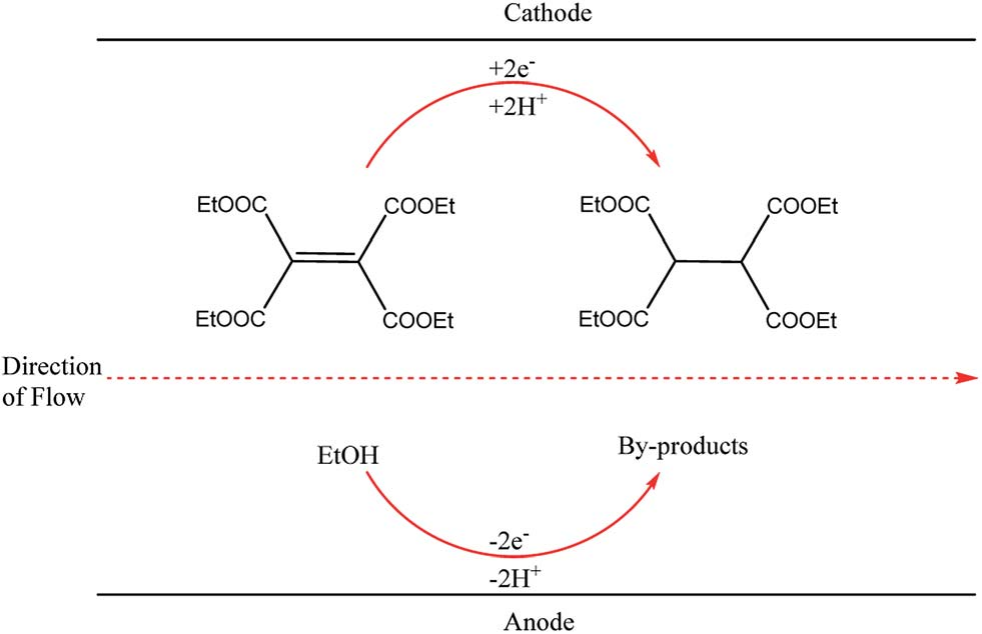
Hydrogenation of tetraethyl ethylene tetracarboxylate in ethanol proceeding through the Marken parallel plate-to-plate microreactor in the absence of a supporting electrolyte.

This finding inspired other researchers to develop self-supported electrosynthetic processes using a flow microreactor without intentionally adding supporting electrolytes. An example of a parallel plate-to-plate an electrochemical flow microreactor in which the addition of a supporting electrolyte is not necessary was reported three years later by Marken *et al.*^28^ A short distance (of 50 μm) between the electrodes aided the conductivity of the medium and also allowed the diffusion layers of the anode and cathode electrodes to overlap or couple, resulting in the formation of ions between the electrodes which then act as the supporting electrolytes. Their reduction of tetraethyl ethylene tetracarboxylate in ethanol afforded the product tetraethylethanetetracarboxylate in yields up to 92% (Fig. 2).

### 2.2. Reactors with compact configuration

**Fig. 3:**
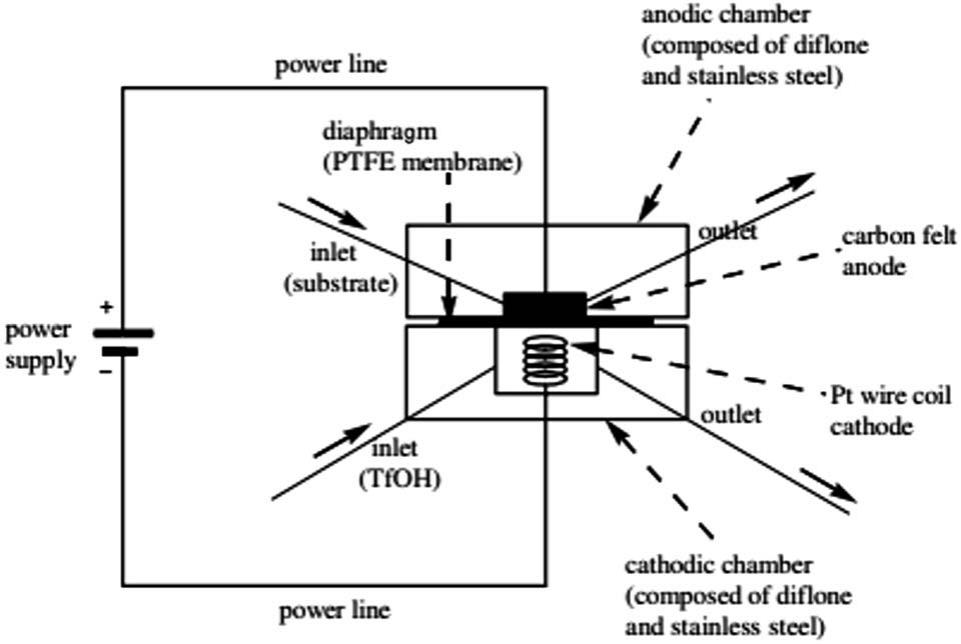
Schematic illustration of the first generation Yoshida electrochemical microreactor. Reproduced with permission from ref. 29, American Chemical Society.

There has been a lot of work performed by Yoshida and co-workers to do with the design of a new thin-gap, compacted microfluidic device for electro organic synthesises.^29^ One of their original designs (shown in Fig. 3) consisted of two diflone and stainless steel bodies, separated by a PTFE membrane. To monitor the “cation flow”, a low-temperature flow cell with a Fourier transform infrared (FTIR) spectrometer was attached to the outlet. The system was applied to oxidative C–C bond formation of various carbon nucleophiles, in which a range of percentage conversions and selectivity's were obtained.

**Fig. 4:**
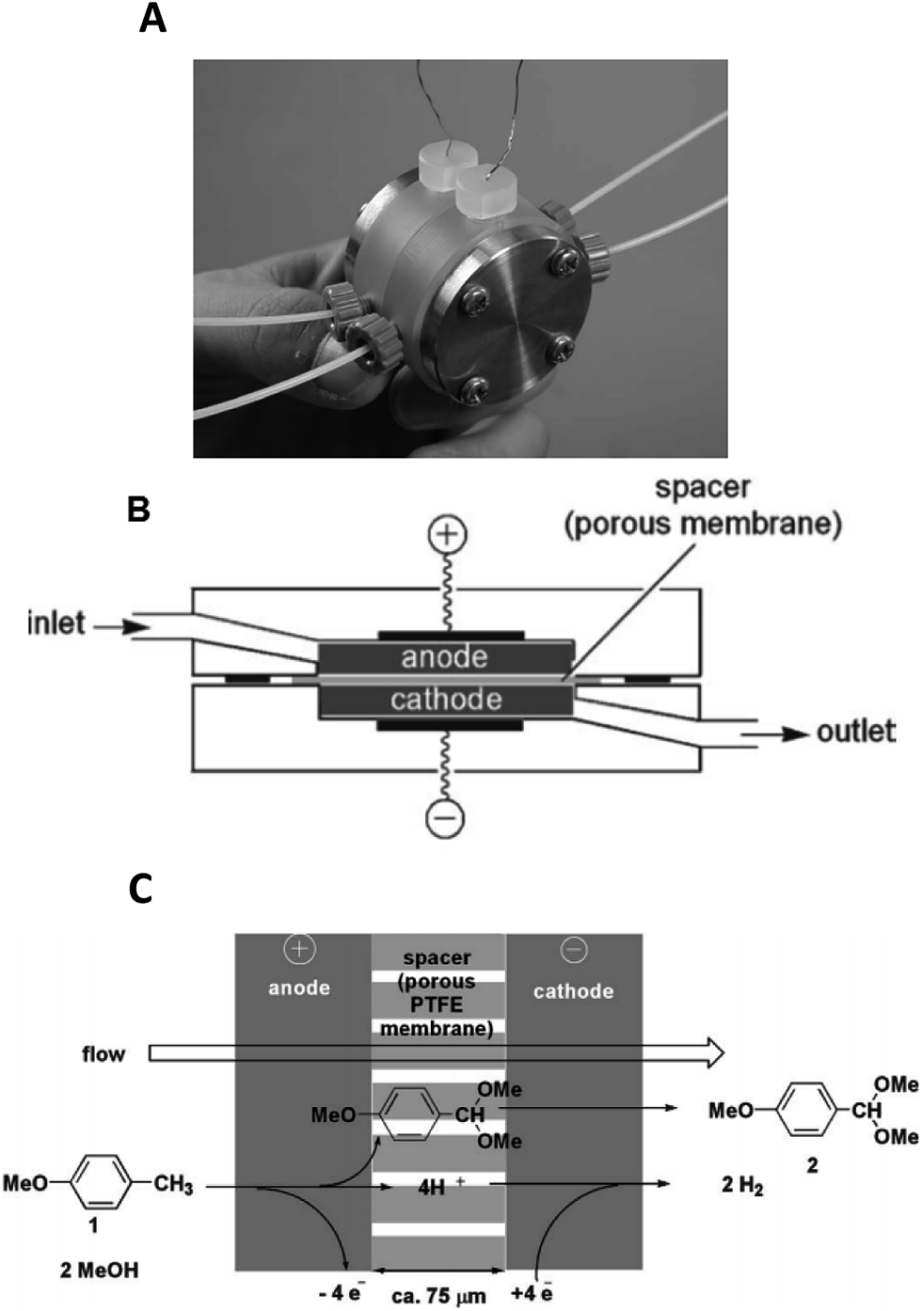
Monochromatic photograph of the electrolyte-free microreactor (A), its schematic representation (B) and the electrochemical reaction scheme for the corresponding oxidation of *p*-methoxytoluene in methanol (C). Reproduced with permission from ref. 30, the Royal Society of Chemistry.

Yoshida *et al.* furthered this technological idea into a microsystem that could operate electrosynthesis in an electrolyte-free environment.^30^ The new reactor comprised a 75 μm thick, porous PTFE spacer (diameter = 20 mm, pores = 3 μm) within two carbon fibre electrodes (Fig. 4). This design contrasts with the aforementioned electrolyte-free Marken reactor,^28^ developed three years earlier, in a number of aspects. Firstly, the flow of the solution passes through the anodic chamber, the spacer and then out through the cathode, hence the flow of solution and electric current are parallel to each other rather than perpendicular. Secondly, the chamber is filled with a carbon felt electrode (to fill the once empty space), resulting in a much larger surface area. Finally, a higher current and flow rate are allowed. This system was then applied to the industrially important anodic methoxylation of *p*-methoxytoluene. At the very small inter-electrode distance, protons could act as the charge carriers, ultimately affording the desired methoxylated product in more than 90% yield.

**Scheme 1:**
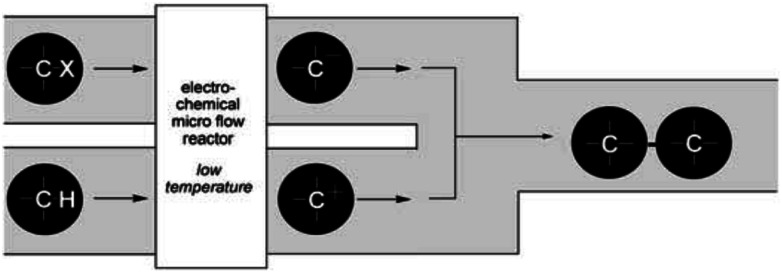
Cation flow method showing the generation of organic ions after passing through a microreactor. Reproduced with permission from ref. 31, Wiley.

Their reactors, despite being limited by low flow rates and hence low product formations, have been used in several studied based upon their concept of “cation flow” (Scheme 1), where organic cations are generated at low temperatures and then reacted with nucleophiles as soon as they leave the microreactor.^31–34^ Extending this cation flow idea, an evolved cell was fabricated that involved a paired microflow system to simultaneously generated organic anions and cations which would then couple with one another *via* simplistic C–C bond formation.

**Fig. 5:**
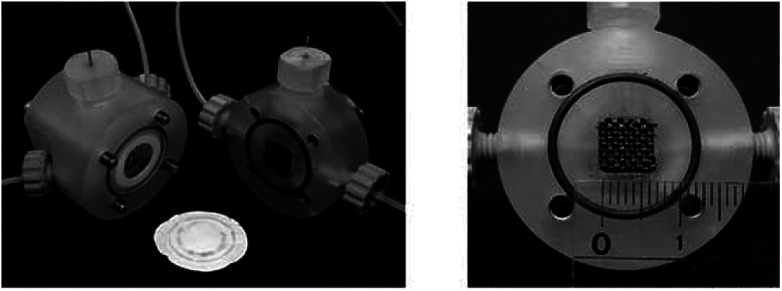
Yoshida and co-workers electrochemical microreactor with carbon felt mesh (7 mm × 7 mm × 5 mm) made from carbon fibre, separated by a PTFE membrane (pore size 0.1 μm). Reproduced with permission from ref. 31, Wiley.

**Fig. 6:**
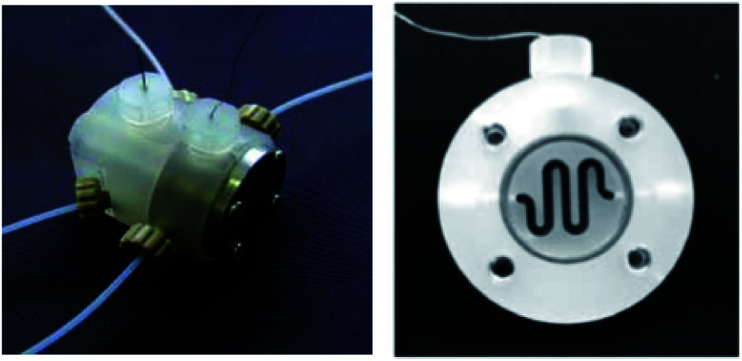
Photograph of the fixed compact micro-device (left) and its cross-section showing winding channel (right): width 1.5 mm, depth 4 mm, length 57 mm. Reproduced with permission from ref. 31, Wiley.

**Scheme 2:**
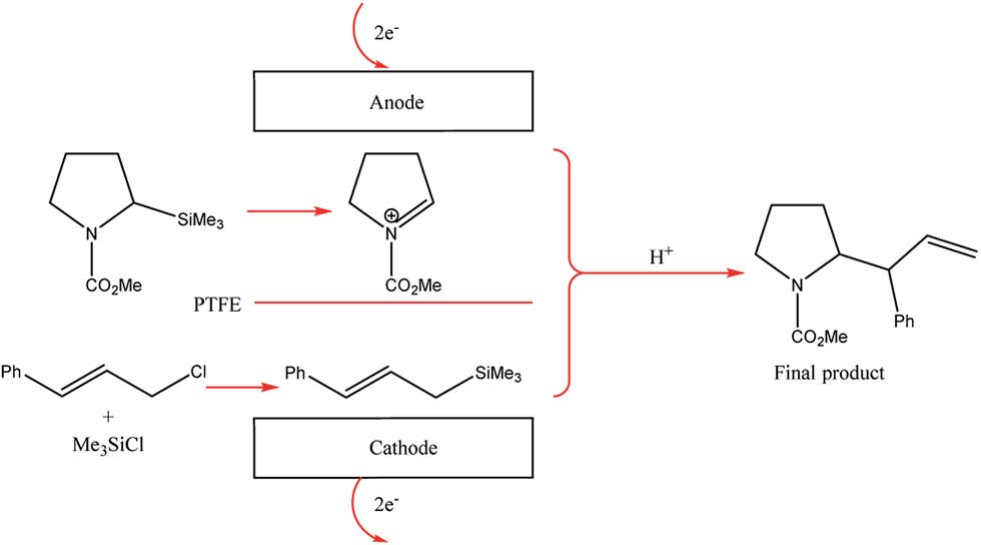
Paired electrolysis of silylsubstituted carbamate and cinnamylchloride in the compact microreactor.

The two-compartment microreactor now consisted of diflone and stainless steel bodies (Fig. 5) with a PTFE membrane dividing the electrode chambers. The cathode compartment was supplied with a 5 cm platinum wire coil, while the anode compartment had a mesh-like microstructured carbon felt anode (7 mm × 7 mm × 5 mm, *ϕ* 10 mm). The whole apparatus was then dipped in a dry-ice bath. Conversions of 49–69% for nitrogen-containing methyl carboxylates with unsaturated trimethylsilicon compounds afforded the carbamate coupling products in 67–100% yields. However, for a reasonable conversion, this reactor with its rectangular compartment required a considerable amount of excess electricity (*ca.* 5 F mol^−1^). The anode compartment was later modified in order to possess a snaking channel of length 57 mm, filled with carbon fibre (Fig. 6). After which the devices performance and current efficiency were significantly improved. To test the microreactor, the concurrent oxidation of a silylsubstituted carbamate, and reduction of cinnamylchloride, in the presence of chlorotrimethylsilane afforded an *N*-acyliminium ion and cinnamyltrimethylsilane (75% yield), that then coupled together to generate the final product, in good yield (79%) with a 85% conversion from starting material (Scheme 2). Mode of operation used was constant current.

### 2.3. Reactor with flux module configuration

**Fig. 7:**
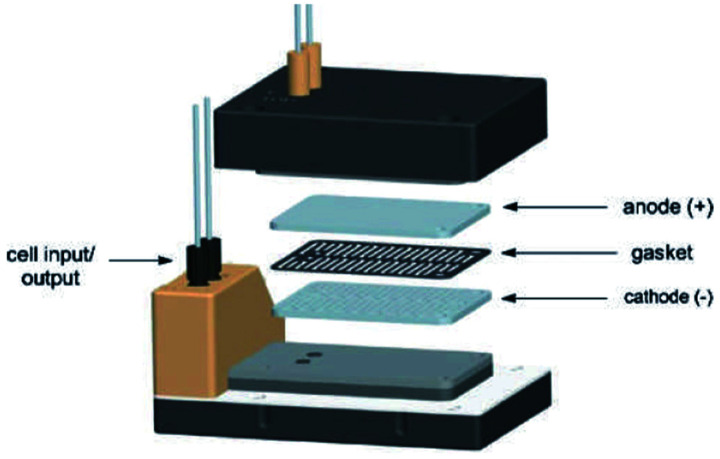
Pictorially described flux module system, developed by Roth *et al.* Reproduced with permission from ref. 36, Academiai Kiado.

**Scheme 3:**
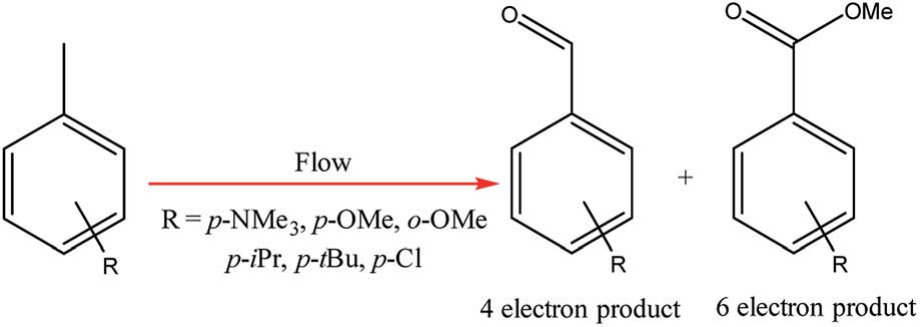
Electrochemical methoxylation/oxidation of substituted toluene derivatives.

Electrochemical oxidations performed in batch are subject to the problem of over-oxidation since high degrees of chemical conversions often require an extension in reaction times. This issue can, however, be combated by the use of flow micro-technology^35^ because desired products can be continuously removed shortly after they have been generated, and replaced with new starting materials.^36^ Thus, the Roth and co-workers gained motivation to manufacture an electrochemical flux module microreactor^36*a*^ to evaluate the functionality of flow-assisted four- and six-electron benzylic electrooxidations from substituted toluenes. The design of this microfluidic cell is a modular plate-based, multiple input system, able to withstand pressures of up to 6.5 bar and an operating temperature range of 0 to 65 °C (Fig. 7). The base of the module has been imparted with a resistance temperature detector (RTD) and a multi-pin electrical lead for convenient maintenance and disassembly. The authors employed a constant current mode of operation, utilising a variety of electrode compositions ranging from carbon fluoropolymer (PVDF) hybrids to stainless steel and platinum-plated; found the C/Pt combination as the set with the highest yielding performances. A range of substrates were subject to optimisation control experiments, possible with good reproducibility, with the six-electron oxidation of *p*-methoxytoluene affording the highest yield reported of 62% (Scheme 3). It must be noted, however, that no one single protocol was possible in order to achieve optimal performance for oxidations across a variety of electron-rich and electron-deficient aromatic substrates. Therefore, each individual substrate will require its own optimisation to ensure a high yielding transformation. It was also revealed that if the oxidation potential of a substrate is too low, a limiting factor of over-oxidation inhibits efficient conversion. Flipping over to the other side of the coin, oxidation potentials exceeding 2.3 V achieved little or no methoxylation.

### 2.4. Reactor with copper plate configuration

Two electrochemical microreactors^37^ for the selective generation of copper–N-heterocyclic carbine complexes from imidazolium precursors have been developed by Chapman *et al.* The first consisted of two parallel copper plate electrodes separated by a PTFE spacer with a linear flow channel (19 cm long by 4 mm wide). The separation of the electrodes was, however, quite long at 2.5 mm, which ultimately contributed to the formation of unwanted side products, along with poor mixing. To reduce the volume within the flow channel, increase flow viscosity, improve selectivity and residence time, glass beads (diameter = 2 mm) were packed into the channel. Now, with an applied voltage of 2.5 V and flow rate of 0.5 mL min^−1^, maximum conversion of the desired product was achieved in single-pass (36% in 120 s) and recirculation mode (92%, 80 min).

**Fig. 8:**
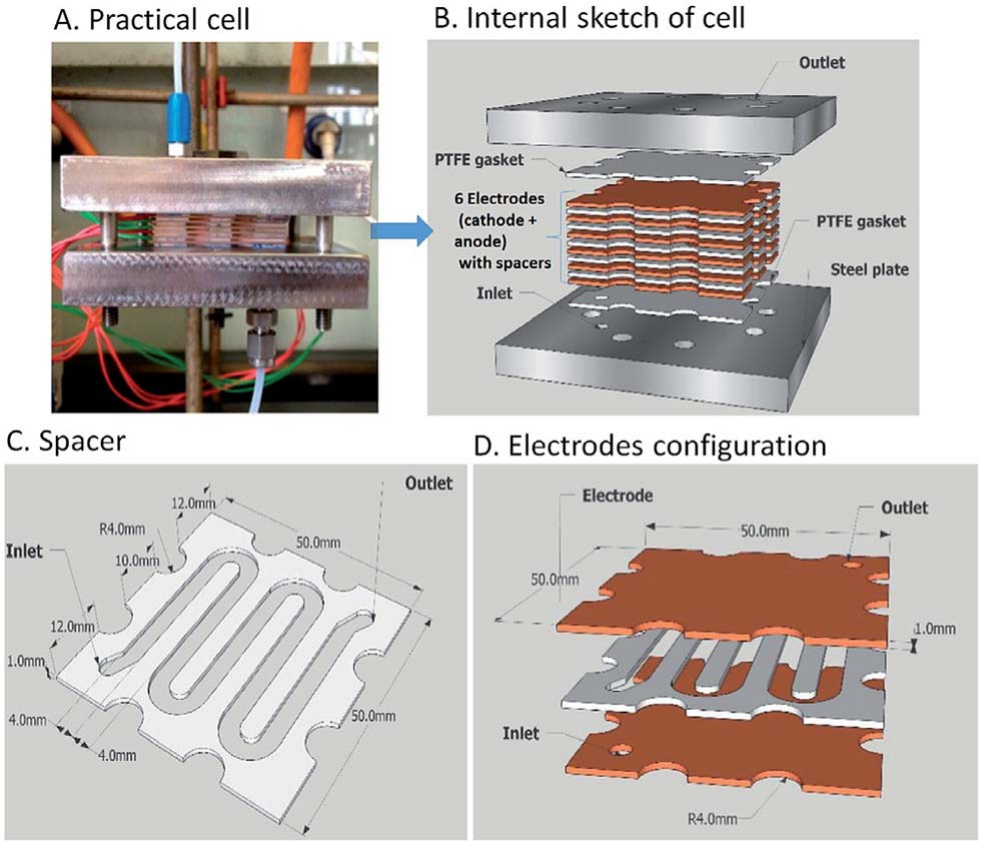
The electrochemical flow-reactor (A and B), the reactor channel through a 1 mm thick Teflon spacer (C) and Cu electrodes configuration (D). Figures reproduced with permission from the ref. 37, the Royal Society of Chemistry.

An improvement upon this design was established by incorporating a stack of six copper electrodes (5 × 5 cm) with five PTFE spacers between them. A monopolar electrical connection gave alternating anode and cathode plates providing five consecutive parallel plate electrochemical flow-reactors and the separation was reduced to 1 mm (Fig. 8). The flow channel was revised into a snaking pathway with five interelectrode gaps to assist the flow movement between plates to reveal a flow channel of length 20 cm and width of 4 mm. For reaction optimisation, reactor parameters (electrode separation, interfacial area, volume channel length and width) could be altered. In this second reactor the cell voltage was lowered and product selectivity was improved. At a potential of 1.94 V and flow rate of 0.67 mL min^−1^, 94% of the product was formed in single-pass mode with a residence time of 360 seconds. Converting 0.132 mmol of starting material into product *via* single-pass mode was achieved in 29.9 minutes with a yield of 97%.

### 2.5. Reactors with chip-type configuration

**Fig. 9:**
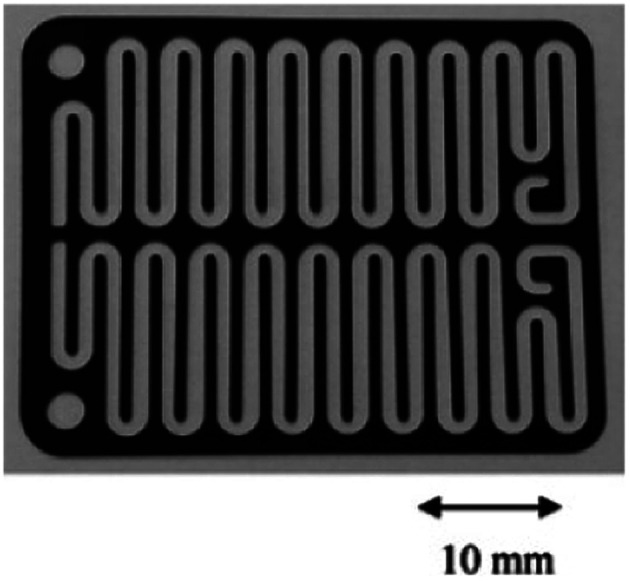
Photograph of the FFKM spacer with snaking channel. Reproduced with permission from ref. 38, Elsevier.

Chip-type electrochemical flow microreactors are a type of plate-to plate configuration and have become popular, particularly for analytical purposes. However, due to their small size and productivity they are generally less suitable for synthesis. An electrochemical flow microreactor has been created for the methoxylation of *N*-formylpyrrolidine. Appropriate chemistry afforded high conversions (up to almost 100%) with desirable reaction rates, selectivity and current to form the product in high purity without any organic side products. The cell itself consisted of two rectangular electrode pates (53 mm × 40 mm × 2 mm thick) with 1050 mm^2^ per electrode surface area in contact with the solution. The cathode was made from stainless steel and the anode from carbon filled polyvinylidene fluoride (PVDF), separated from each other by a perfluoroelastomer (FFKM) spacer. The spacer had a snaking microchannel cut into it (depth and width of ∼200 μm and 1.5 mm respectively) which therefore, allows a longer channel (length 700 nm) to be in contact with the electrodes (Fig. 9).^38^

**Fig. 10:**
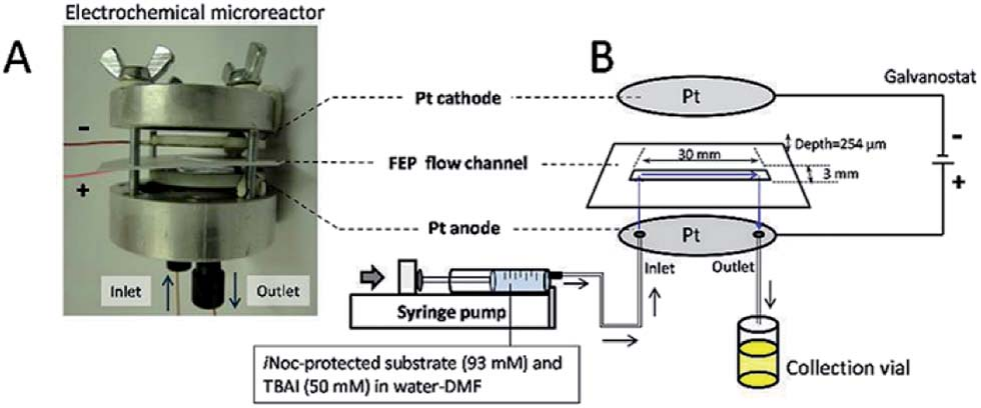
(A) Photograph of the opened electrochemical microreactor and (B) schematic illustration of the flow setup for the electrochemical deprotection of the ^i^Noc group. Reproduced with permission from ref. 39, Beilstein-Institut.

**Fig. 11:**
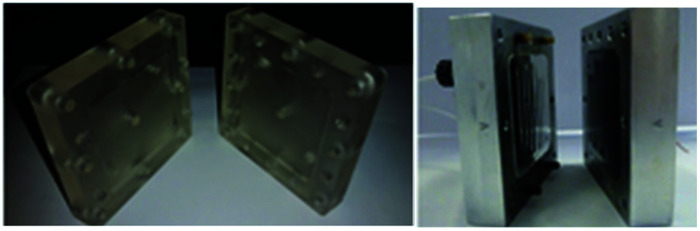
Electrochemical flow 3D printed reactor (left). Electrochemical flow reactor with aluminium body (right). Reproduced with permission from ref. 4, Wiley.

Contributing to this development, Wirth and co-workers^39^ designed a simple electrochemical microreactor consisting of two aluminium bodies (50 mm diameter, 25 mm height) attached to two polytetrafluoroethylene (PTFE) plates with platinum electrodes (0.1 mm) sandwiching a fluorinated ethylene propylene (FET) separator through which the reaction solution flows through (Fig. 10). This system is advantageous in the sense that it has large electrode areas (25 cm^2^ each), is easily assembled and dismantled, and the material of the electrodes can be easily exchanged. This gives the system greater flexibility, efficiency and productivity. When applying this technology to the synthesis of diaryl iodonium salts, purification was unnecessary as the product salts precipitated with potassium iodide in yields of 18–72%. In this case, H_2_SO_4_ acted as an electrolyte between the electrodes and as the counter ion for diaryl iodonium hydrogen sulfates (intermediate).^39^ Their yields of the Kolbe electrolysis reactions of di- and trifluoroacetic acids in the presence of various electron-deficient alkenes was comparable, and in some cases higher, than that of a batch reactor (batch: 11–45%, flow: 11–52%),^40^ and in another work with the same reactor, the deprotection of an ^i^Noc group from phenols and benzenethiol occurred in a rapid fassion.^41^ Alternatively, the reactor can be made from polymers instead of aluminium, by using 3D printing additive manufacturing technology, allowing a reduction in the time and cost of construction as well as simplistic approach to customisation if required (Fig. 11).^4^

**Fig. 12:**
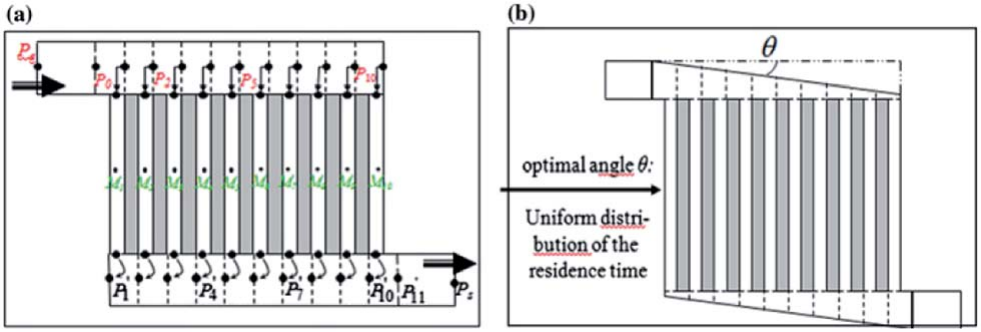
Schematic representation of the Tzedakis *et al.* microstructured electrode incorporating 10 microchannels (length 5 mm and depth 50 μm for microchannels and collecting/distribution channels; widths 250 μm for microchannels and 1 mm for collecting/distributing channels). (a) One possible non-optimised geometry, (b) optimised geometry achieved by changing the opening angle *θ* of the distributing and collecting channels. Reproduced with permission from ref. 43, Springer.

Chip-type microreactors have been constructed with a network of microchannels in an attempt to improve electrochemical reactions. In 2012 Tzedakis *et al.* designed an undivided microreactor to effectively run thermodynamically unfavourable electrosynthesis.^42,43^ The multichannel configuration was equipped with a heat exchanger to either supply heat to endothermic reactions or to rapidly remove heat produced in exothermic reactions. A network structure was also imparted into the design for optimal residence time distributions (Fig. 12).

**Fig. 13:**
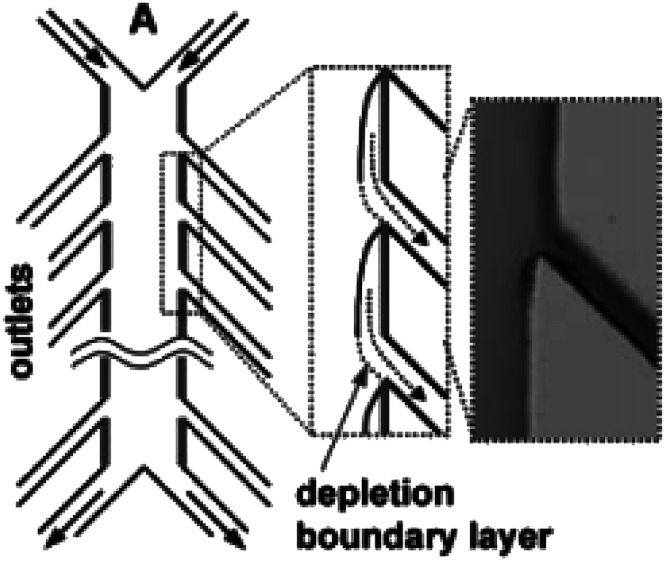
Multiple outlet designed by Kenis *et al.* to periodically remove the depleted zone. Inset: Optical micrograph of removal of a dyed stream. Reproduced with permission from ref. 44, the Royal Society of Chemistry.

Similarly, a Y-shaped microreactor containing sputtered gold electrodes lining the interior of the major channel was reported by Kenis and co-workers.^44^ Here, the reactor performance could be increased from 10% to 100% by the incorporation of with multiple inlets and outlets on polycarbonate sheets (Fig. 13).

### 2.6. Reactors with modular configuration

**Fig. 14:**
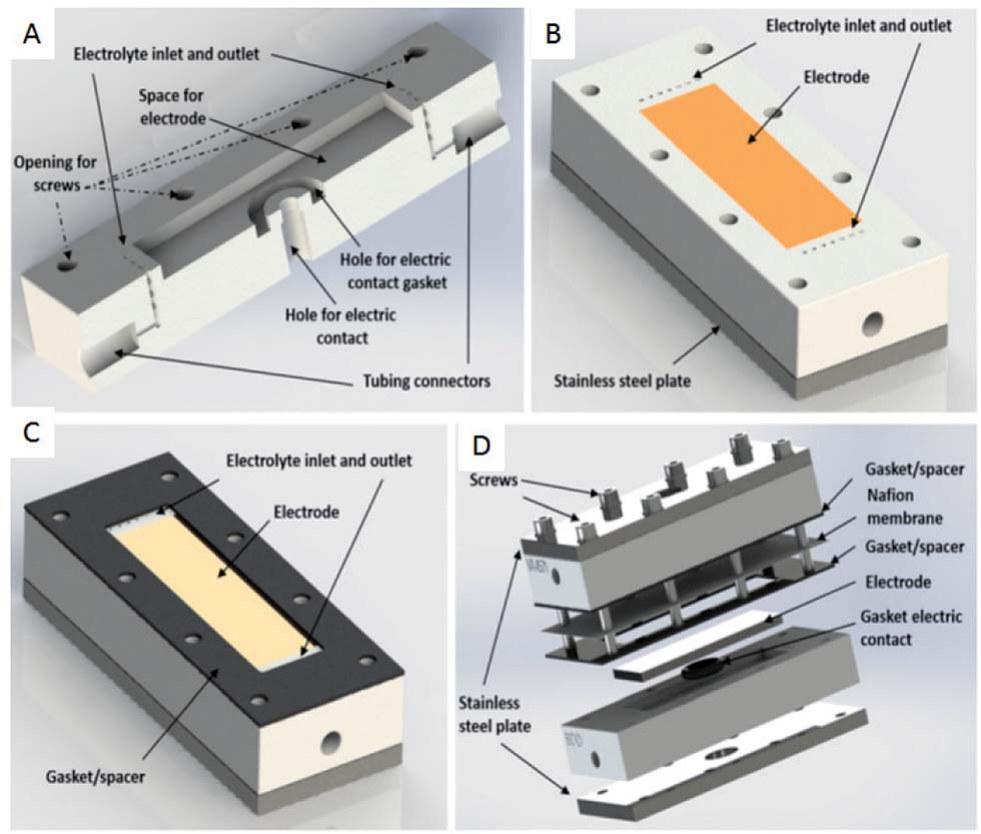
(A) Cross-section of the modular reactor developed by Waldvogel *et al.* displaying the Teflon piece with the connections for tubing, inlet, and outlet and free space for the electrode. (B) Complete half-cell that shows the cavity (dimensions = 60 × 20 mm), the electrode, Teflon piece, and a plate made of stainless steel (C) the same half-cell, this time containing the gasket/spacer on the top. (D) Schematic illustration of the complete divided cell. In the undivided mode, the Nafion membrane and one gasket/spacer are excluded. Reproduced with permission from ref. 45, American Chemical Society.

**Scheme 4:**
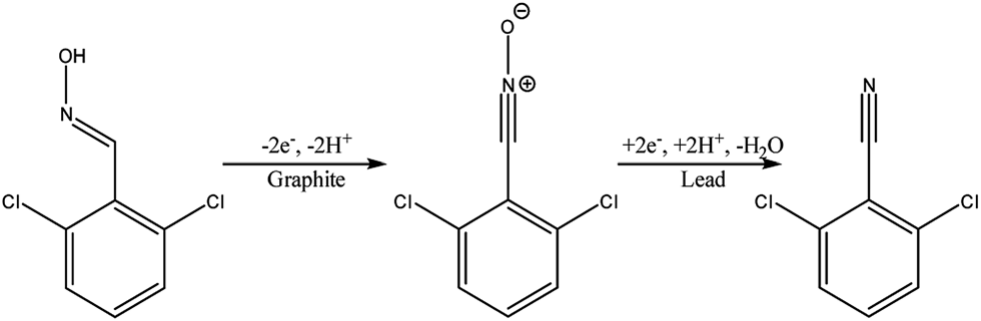
Reaction scheme for the domino oxidation–reduction of the desired nitrile 3 (far right) from oxime 1 (far left).

Another type of microreactor, similar to the modular copper plate reactor described above, was recently reported by Waldvogel and co-workers^45^ of whom have now described a commercially available, highly modular electrochemical flow cell (Fig. 14). The cell is comprised of two Teflon pieces (100 × 40 × 16 mm) with a cavity of 60 × 20 × 3 mm in which to fit an electrode material and to position a power supply connector. The positioning of the 60 × 20 × 3 mm electrode affords a coplanar surface with the Teflon, and the connector is separated from the electrolyte by a gasket/spacer. The applicability of this modular reactor was demonstrated by electrochemical reaction in both a divided and undivided cell setup. As an illustration, in the undivided mode of operation, a domino oxidation-reduction for the synthesis of a nitrile was performed (Scheme 4). Imparting a graphite anode, oxime 1 is oxidised to the corresponding nitrile *N*-oxide, which is then, at a lead cathode, directly reduced to the desired nitrile 3. When nitrile 3 was recrystallised from ethanol/water (2 : 1) at −30 °C, a pure product of 63% yield was obtained (as opposed to its batch counterpart of 41%). Applying a spacer of 0.12 mm, flow rate of 8.5 mL h^−1^ and a current density of 5 mA cm^−2^, a yield of 80% was achieved.

This novel setup ultimately provides the user with a convenient way in which to switch between divided and undivided configurations, easy adjustment of the distance between the electrodes, and the ability to simply change the material of the electrode. The system only uses electricity and recyclable solvents, column chromatography is not required and the need for other (harsh) reagents and supporting electrolytes are unnecessary. Arguably, however, the most beneficial aspect of this cell is the exact thermal positioning of the electrode material into a Teflon piece, allowing the possibility for nonmachinable electrode materials such as glassy carbon or boron-doped diamond to also be applied.

### 2.7. Reactor for polymerisation

**Fig. 15:**
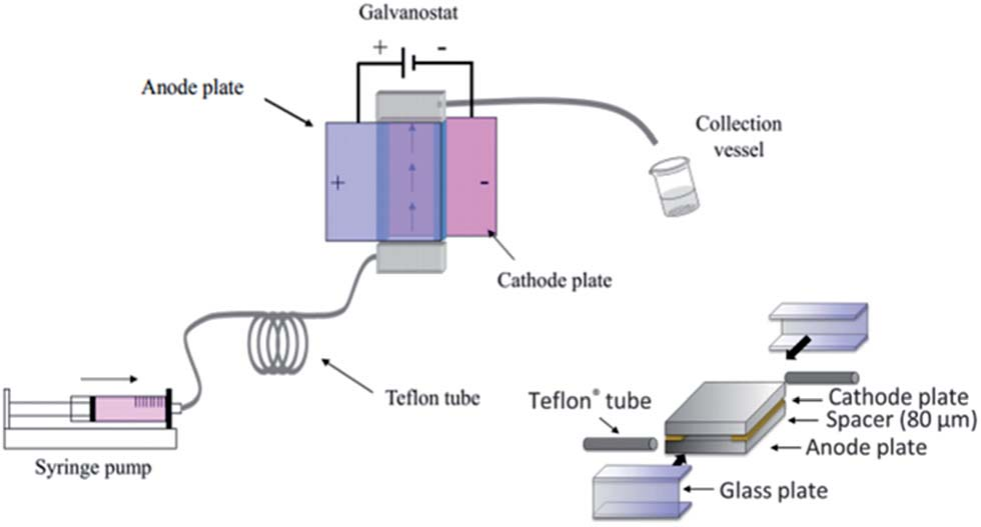
Schematic diagram of the electrochemical flow set-up (left) and construction of the microreactor (right). Reproduced with permission from ref. 49, the Royal Society of Chemistry.

Polymerisation has also been performed in microflow systems.^46–48^ Recently, Atobe *et al.* constructed a microreactor for the electrochemical synthesis and molecular weight control of the π-conjugated poly(3-hexylthiophene) (P3HT) (Fig. 15).^49^ The properties of P3HT are directly influenced by the molecular weight, hence control over this parameter is an important one. Due to its properties, such as processability, environmental stability, charge mobility and wide solubility have made P3HT a promising material for organic photoelectronics (solar cells, electroluminescent devices and field-effect transistors).^50–52^ For controlling molecular weight distributions in P3HT, conventional batch methodology utilises transition metal catalysts for cross-coupling polycondensation.^53,54^ These synthetic methods are, however, limited by the toxicity of the transition metal catalysts, long reaction times, multi-step processes and difficulty in developing continuous large-scale processes. Their flow reactor afforded a much greater monomer conversion (53–68%) compared to the batch reactor (13%). This was attributed to more efficient mixing, which in-turn would allow a more efficient electrode reaction of the monomer in the microreactor. Flow also gave a narrower molecular weight distribution (1.7 in flow, 3.5 in batch) accredited to the microreactors high surface area-to-volume ratio causing the absence of hot spots and therefore, better defined polymer products.

**Scheme 5:**
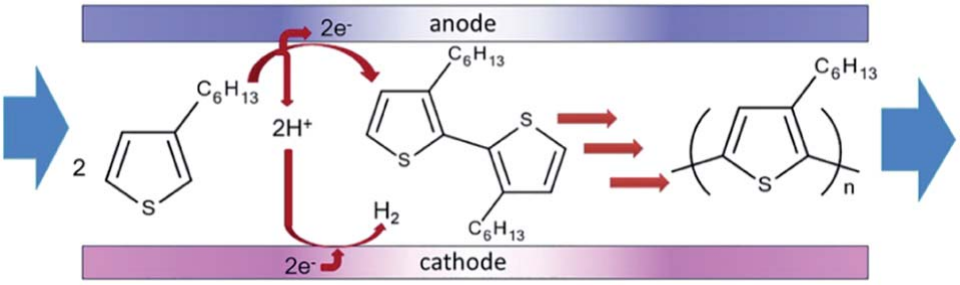
Flow reaction scheme for the electrochemical synthesis of poly(3-hexylthiophene) (P3HT). Reproduced with permission from ref. 49, the Royal Society of Chemistry.

Concurrently, flow can avoid the deposition of the polymer on the surface of the anode, allowing continuous synthesis of the soluble polymer (Scheme 5). The reaction at the cathode is the reduction of protons generated at the anode, therefore, does not affect the process at the anode. Furthermore, screening of the electrochemical flow conditions was used to control the molecular weight of P3HT. Graphite as the anode material gave higher conversion and a narrower polydispersity compared to other anode materials, the most suitable solvent for this system was dichloromethane, and a decrease in flow rate (increase in electricity/F mol^−1^) led to an increase in the polymers molecular weight. In this work an electrolyte was also employed and it was observed that a decrease in donor number of the anion gave an increase in the molecular weight (Bu_4_NPF_6_ found to be optimal). Reasoning has been allocated to an acceleration in the rate of coupling between monomer and oligomer radical cation intermediates by using a smaller donor number anion. The smaller the donor number, the fewer the ion-pair interactions with the radical–cation intermediates.

The microreactor consisted of a stainless cathode plate and an anode made from graphite (or a Pt plate, glassy carbon plate or glass plate coated with ITO). Both electrodes faced one another and were 3 cm by 3 cm in width and length. Between them, a 80 μm thick spacer, double faced adhesive tape, left a rectangular channel (10 mm wide and 30 mm long) sandwiched between the plate electrodes. After connecting Teflon tubing to inlets and outlet, the reactor was then sealed with epoxy resin (Fig. 15).

### 2.8. Reactor with multiple channel configuration

**Fig. 16:**
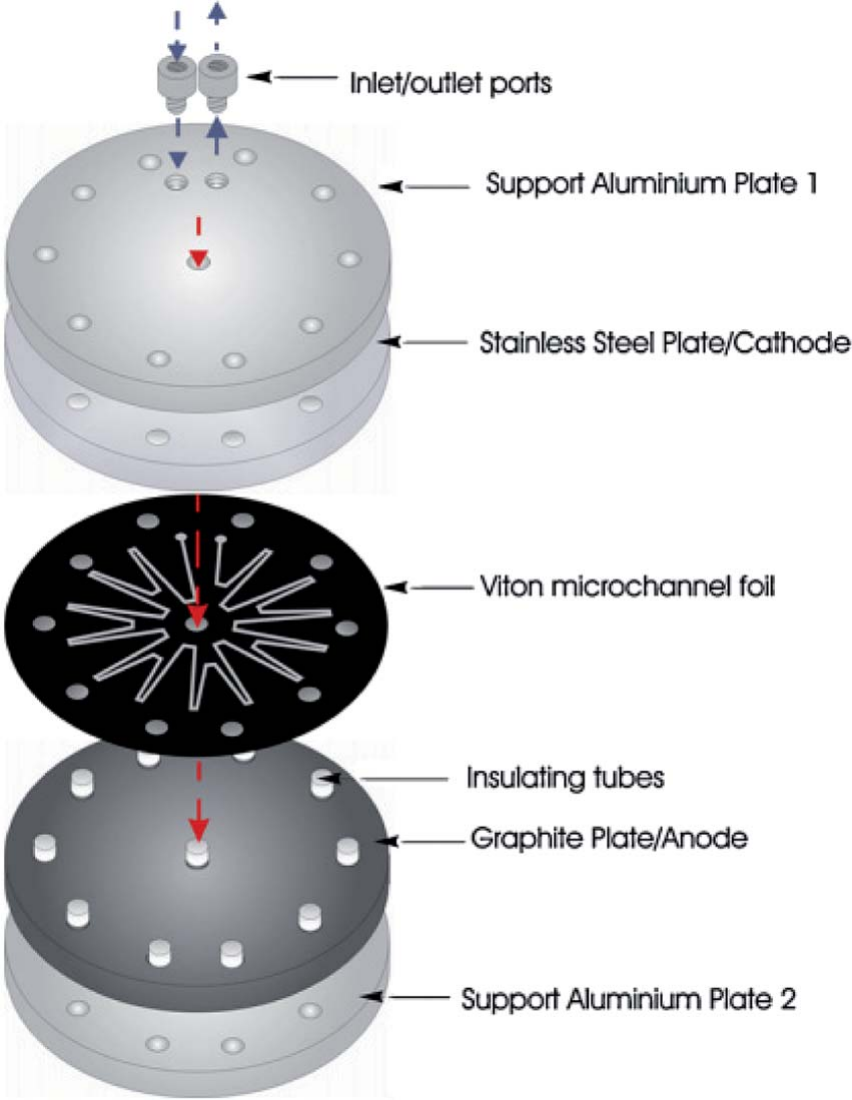
Schematic illustration of the round electrochemical microreactor developed by Birkin and co-workers, showing its essential components. Reproduced with permission from ref. 55, Elsevier.

Birkin and co-workers^55^ have also developed an inexpensive and simple electrochemical microreactor for organic synthesis, reporting that the device could perform with a high degree of product formation and selectivity in a single pass. The reactor had two flat electrodes, diameter of 100 mm, separated by an approximately 500 μm thick by 1 mm wide, lasercut starburst-shaped fluoropolymer elastomer (Viton) spacer, consisting of several sharp turns to reveal a total channel length of 600 mm (Fig. 16). The round design was employed to give more evenly distributed pressure across the plates and therefore, prevent leakage. The cathode was made from stainless steel (3 mm thick) and the anode form a conductive carbon/polyvinylidene fluoride (PVDF) composite (5 mm thick). Two aluminium plates sandwiched the electrodes, they were sealed with eleven stainless steel bolts to help prevent electrolyte leakage from the cell, and two aluminium connectors (to the commercially available flow system [FRX, Syrris]) were attached to the inlet and outlet positions.

**Scheme 6:**
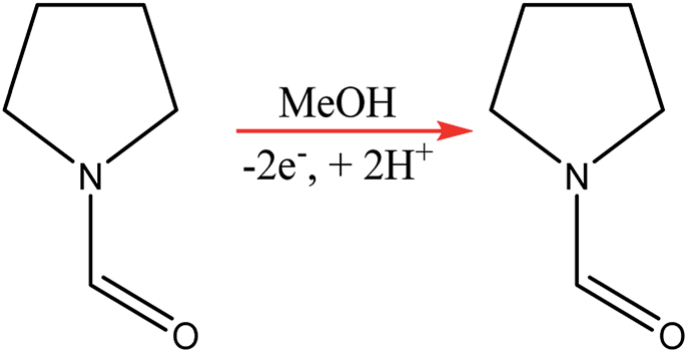
Methoxylation of *N*-formylpyrrolidine.

Using the anodic methoxylation of *N*-formylpyrrolidine as an example reaction, since it is known that they give good yields and selectivity in parallel plate cells,^56^ optimisation was able to afford conversions of up to 96%, with an isolated yield of 87% for the starting material (Scheme 6).

### 2.9. Reactor with fuel cell-type configuration

A great deal of study has been dedicated to miniaturised machines for fuel cell technological applications,^57–59^ enabling an onset of unique, miniaturised and conventionally improved energy conversion units. Such examples include, but are not limited to, microreactors,^60^ micro fuel cells,^61^ micro batteries,^62–64^ miniaturised gas turbines,^65–67^ MEMS piezoelectric,^68^ thermoelectric and electromagnetic power generators,^13,14^ miniaturised heat engines,^69–73^ micro super capacitors,^74^ streaming potential through a nanochannel,^75–85^ and biologically inspired methodology.^86–89^ However, when it comes to large scale production, these micro machines face the complication of expenditure, complex fabrication and difficulties relating to packaging. Hence, these technologies targeted for small scale portable applications such as mobile phones/electronics and laptop batteries. Micro fuel cells and microreactors are devices that are also aimed at achieving portable power for mobile electronics. These devices can successfully convert chemical and biochemical energy into electrical energy. Relating this concept directly to the world of chemistry, Wouters *et al.*^90^ fabricated a fuel cell-type microreactor, based on a design developed by Ferrigno and co-workers,^91^ called the colaminar flow cell (CLFC). Two reactions were performed while producing a small amount of electricity; the reduction of nitrobenzene at the cathode and the oxidation of methanol at the anode. At a flow rate of 55 L min^−1^ and nitrobenzene concentration of 0.0375 M, the highest obtained power density was 0.542 mW cm^−2^. Using a load of 1000 Ω, flow rate of 5 L min^−1^ and concentration of 0.025 M, the average power density was 0.062 mW cm^−2^ while achieving 37% conversion.

**Fig. 17:**
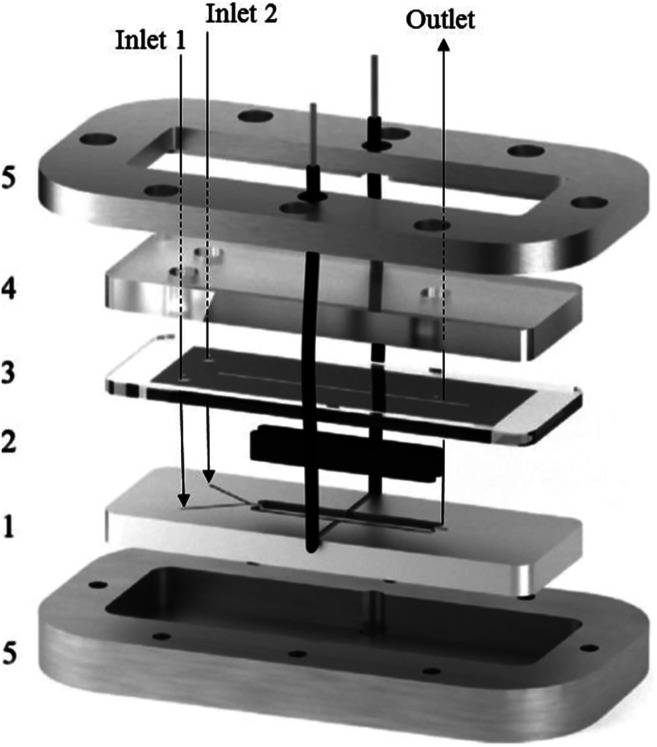
Exploded view of the fuel cell-type colaminar flow cell (CLFC) fabricated by Wouters and co-workers;^90^ (1) Y-shaped flow channel (two smaller channels: 0.5 mm × 0.5 mm × 10 mm, main channel: 1.0 mm × 0.5 mm × 30 mm) cut into a poly(oxymethylene) piece, (2) graphite electrodes coated in catalyst ink (surface area 4 × 28 mm^2^) placed in the polyoxymethylene piece and glued to the electrical wires with silver epoxy, (3) copolymer plate and rubber seal covering the channel, (4) transparent poly(methyl methacrylate) piece with holes for inserting nanoports and tubing, and (5) aluminium plates that hold the device together. Reproduced with permission from ref. 90, Elsevier.

The construction of the fuel cell is described in Fig. 17. The flow channels and electrode compartments are dug into a Delrin® polyoxymethylene (POM) piece (1). The graphite electrodes are coated with catalyst ink and are glued to electrical wires with silver epoxy glue (Circuitworks® CW2400). The flow channel consists of two intersecting channels (dimensions = 0.5 × 0.5 × 10 mm) in a Y-shape (30° angle) joining on to a main channel (1 × 0.5 × 30 mm) with 28 mm long electrodes at each side (2). There is one exit in the main channel, the top of which is closed by a Topas® cyclic olefin copolymer (COC) cover and a rubber seal to prevent leakage (3). The flow is allowed to pass through *via* three small perforations in the inlets and outlet. A polymethylmethacrylate (PMMA) plate covers this seal, and also contains threads for inserting nanoports (4). Both the cover and the top plate are made from transparent polymers to allow the user to be able to look inside the reactor. Finally, all the different pieces of the cell are held together by two pieces of aluminium, screwed together (5). This microfluidic fuel cell uses features of laminar flow to form a stable interface between electrodes, which disregards the need for an expensive semi-permeable membrane. Thus, cost, simple water control, convent assemble and electrolyte pH flexibility are all added advantages of the design.^90^ Furthermore, the flexibility of the pH constraint enables separate alteration of the anolyte and the catholyte, allowing an increase in cell performance, potential difference and compatibility with acidic and basic supporting electrolyte solutions. Finally, during the methanol oxidation self-poisoning by CO occurs, however, at a particular coverage of CO, the anode was found to be able to undergo self-regeneration.

### 2.10. Reactors with filter press type configuration

**Fig. 18:**
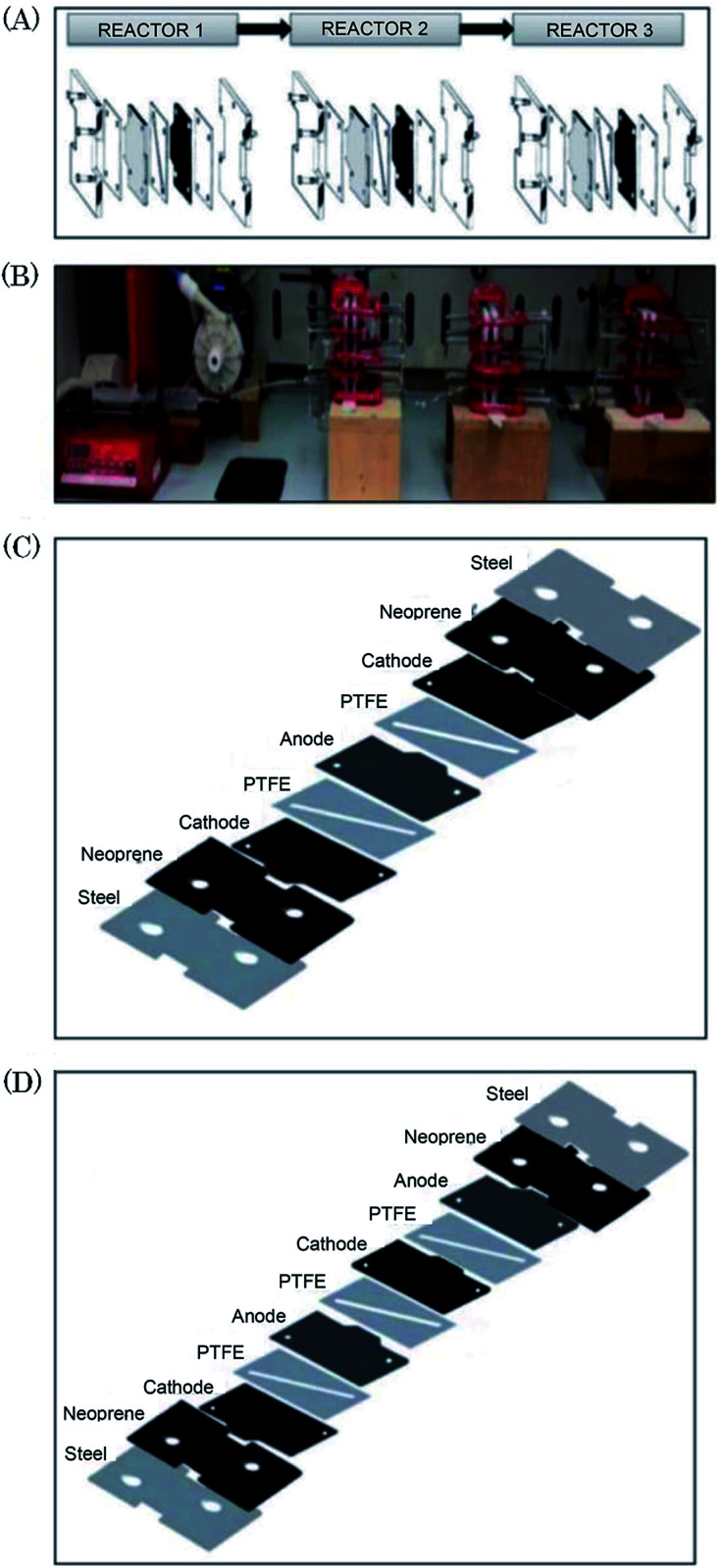
(A) Schematic representation of three microreactors in series, (B) photographic showing the three microreactors in series connected to a syringe pump as used by Scialdone *et al.* (C and D) Schematic illustrations of stacks with (C) two and (D) three cells. Reproduced with permission from ref. 93, Wiley.

Scialdone *et al.*^92^ reported the cathodic reduction of dichloroacetic acid chloroacetic acid in water in two different filter-press electrochemical microreactors. One was constructed with an adhesive spacer, and the other was fabricated using a PTFE micrometric spacer, the later having the added advantage of simple assembly and tolerance for a large range of solvents and electrodes. Both cells were equipped with compact graphite cathode and, under optimised conditions, afforded the product in good selectivity, with high conversion in a single pass mode in the absence of an additional electrolyte, yet at low cell potential. However, productivity and final recovered product of the microreactor was hindered due to the small electrode surface areas (4 cm^2^). Therefore, scaling-up this system was necessary in order to determine its potential practicality. In response to this, the same group performed the same reaction, this time in three different microreactors. A simple cell, a stack containing several electrode chambers, and three cells in series.^93^ Again, electrolyses were performed with the aforementioned benefits, with graphite as the cathode material and Ti/IrO_2_–Ta_2_O_5_ as the anode material. Inter-electrode distance was 0.1 mm and the electrode surface area remained 4 cm^2^. Optimization of the productivity and of the final concentration of the target product is achieved by using a stack with two or three electrode chambers in series. Utilisation of three microreactors in series introduces the possibility to modulate current density among the reactors in order to achieve maximum operating conditions. The stack of several cells in series was fabricated in the manner depicted in Fig. 18, constructed with a commercial undivided filter-press flow cell with three or four electrodes, depending on the number of electrolytic chambers. This enabled a significant increase in productivity (up to 3.1 mmol h^−1^), selectivity (almost 100%), yields (85–97%), and operation at higher initial concentrations of substrates (0.1–0.5 M).

### 2.11. Reactors with three-dimensional electrode configuration

**Fig. 19:**
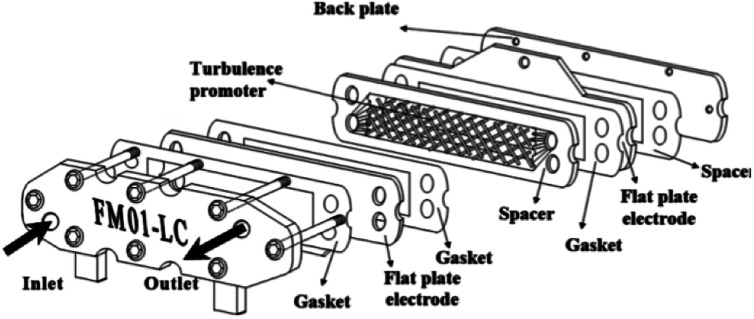
FM01-LC electrolyser with major components separated. Reproduced from ref. 3, American Chemical Society.

The FM01-LC electrolyser (active area of 4 × 16 cm) is a commercial, laboratory-scale cell that was used for many academic studies and for a number of years was marketed by ICI (now AkzoNobel) (Fig. 19).^94–100^ It was based upon the FM21-SP electrolyser (2100 cm^2^), originally been designed for chlor-alkali processing.^99^ The reactor was employed to screening chemical reactions in parallel plate flow cells.

The FM01-LC cell (Fig. 19) is designed in a plate-and-frame-press configuration, containing electrodes, gaskets, ion-exchange membranes and spacers (it could be operated with or without a separator), compressed between two electrically insulating plates. The electrode area could be easily increased by building up a series of standard sized electrode plates or by using additional stacks.

**Fig. 20:**
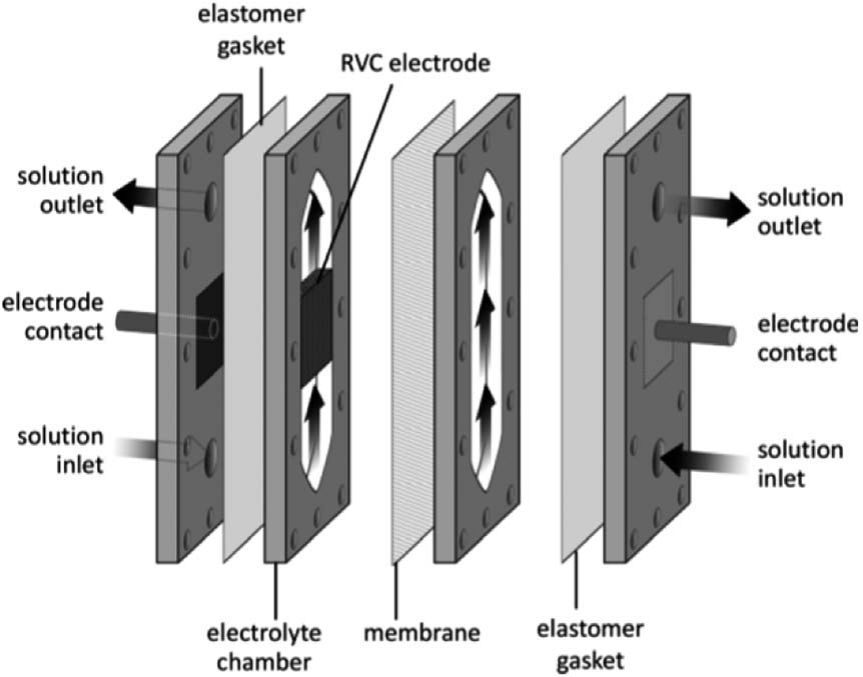
Three dimension foam electrode membrane separator to illustrate a polymer block solution in a flow electrochemical cell. Reproduced with permission from ref. 3, American Chemical Society.

Its configuration allowed the rate of conversion of reactant to product to increases with flow rate^101^ (as a consequence, resulted in a lower fractional conversion per pass of solution). The conversion rate could then be further increased by implementing one or more thin polymer meshes into the solution flow,^101^ with the largest increase being achieved with a three-dimensional electrode, usually a foam, a felt, or a stack of mesh electrodes.^96,97,101^ These three-dimensional electrodes (schematically represented in Fig. 20) are easily incorporated into parallel plate microreactors by adding polymer blocks as the electrode compartments and have an enhanced surface area.^102^

Three-dimensional electrodes have also been experimented in packed-bed electrochemical reactors in an attempt to scale up the reactors capacity, owing to their high electrode surface area-to-volume ratio and high mass transport characteristics.

**Scheme 7:**
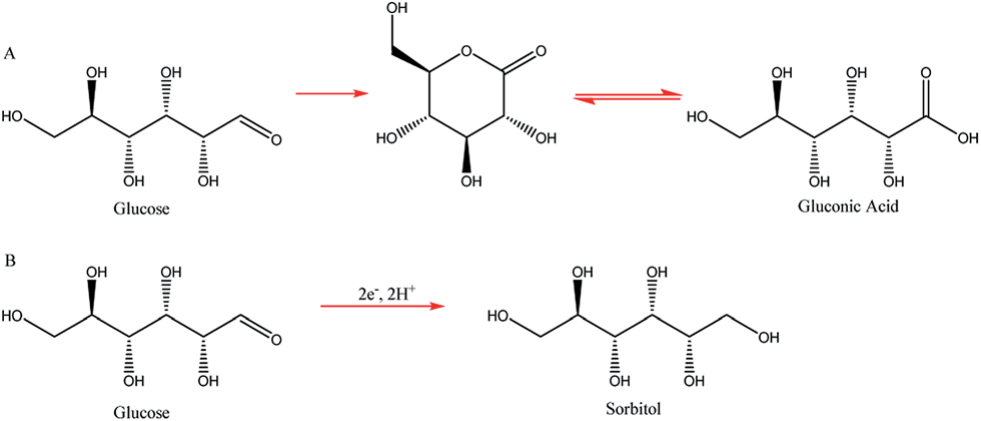
Electrochemical synthesis of gluconic acid (A) and sorbitol (B) form glucose.

**Fig. 21:**
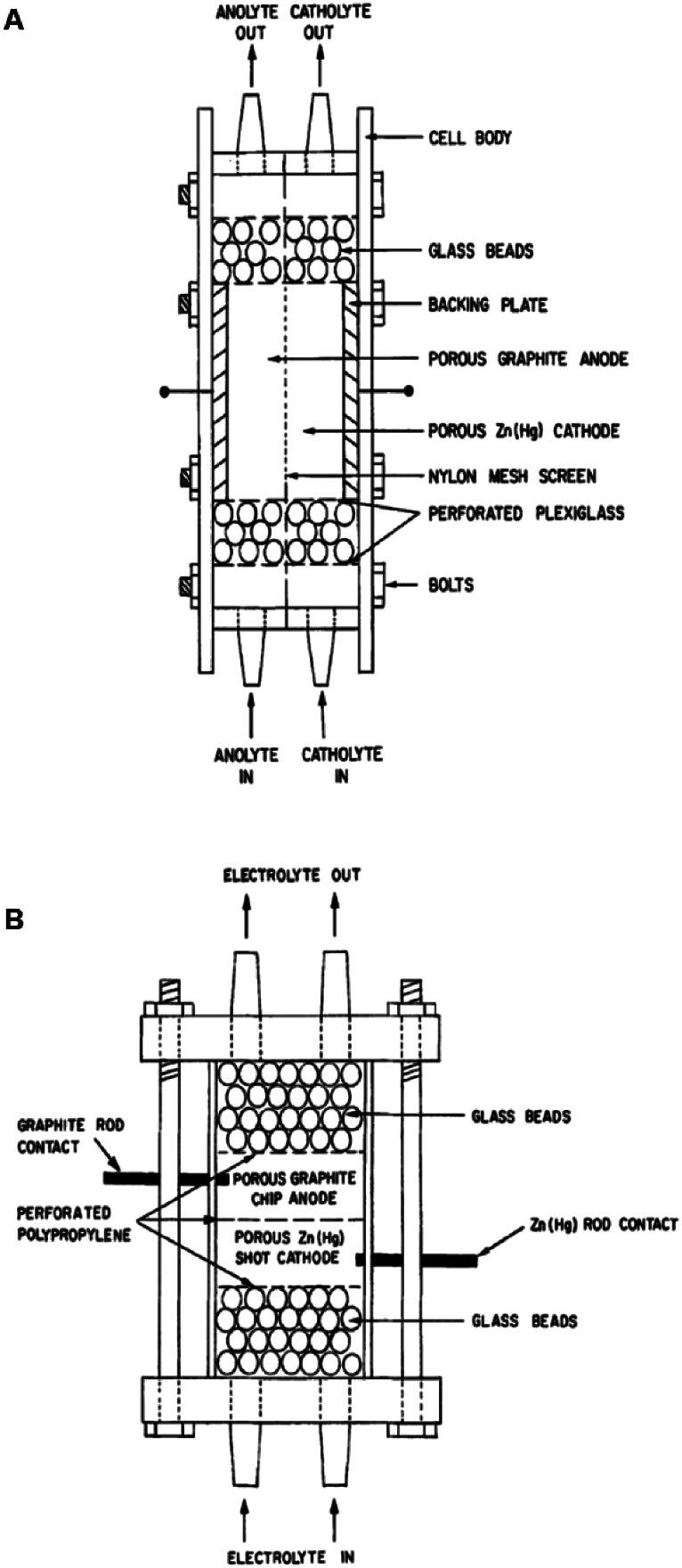
Two packed-bed electrochemical microreactors designed by Nobe *et al.* (A) representing the perpendicular case and (B) the parallel case. Reproduced with permission from ref. 103, Springer.

Two such types of packed-bed reactors were developed by Nobe and co-workers^103^ wherein they applied these devices to the paired electrochemical synthesis of gluconic acid and sorbitol from glucose (Scheme 7). In reactor A, the flow of solution and direction of the current were perpendicular (Fig. 21). A nylon mesh separated the packed beds (dimensions 9 × 3 × 1.5 cm^3^) and glass beads (5 mm) were placed at the entrance and exit to the electrode compartment to allow a uniform flow through the reactor. In reactor B, however, the flow of solution and direction of the current were parallel to each other and the nylon mesh between the electrodes (dimensions 5.7 cm in diameter and 1.2 cm thick) had perforated polypropylene discs held between them. In both cases, the cathode compartments were packed with a zinc shot (0.5 cm) and the anode compartment with cylindrical graphite chips (0.3 cm diameter, 0.3 cm length). The best results for the model reactions were for that of the parallel case in which a maximum current efficiency of 88% and 39% were found for gluconic acid and sorbitol, respectively.

### 2.12. Reactor with C-flow configuration

**Scheme 8:**
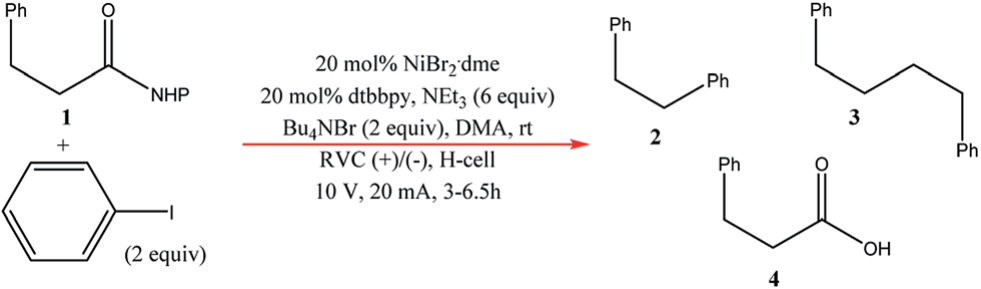
Batch C–C coupling of *N*-hydroxyphthalimide ester with iodobenzene.

**Fig. 22:**
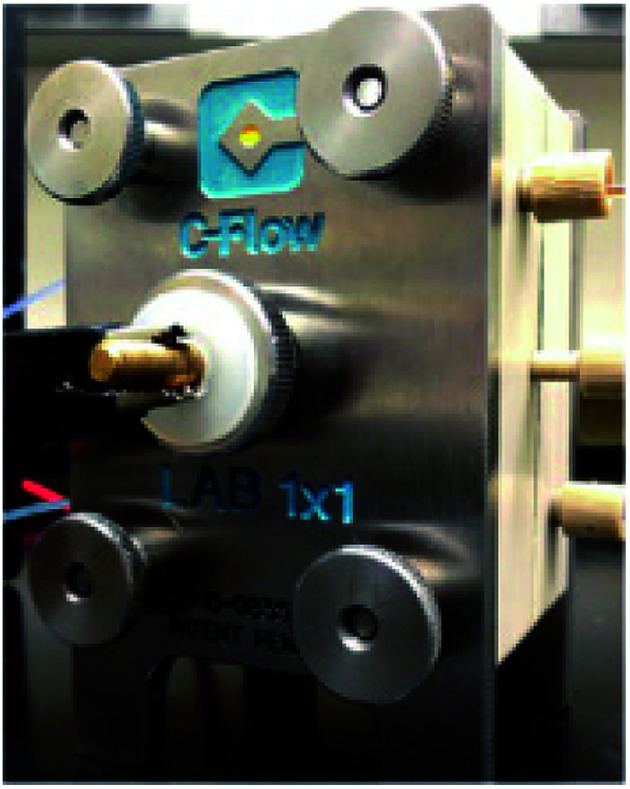
Photograph of the C-Flow LAB 1 × 1 electrochemical cell. Reproduced with permission from ref. 104, American Chemical Society.

Very recently, Bio and co-workers^104^ reported reductive C–C couplings of *N*-hydroxyphthalimide esters with aryl halides in both batch and continuous flow (Scheme 8). Under mild conditions, these reactions were performed with the assistance of a homogeneous nickel catalyst. Under optimised conditions, the coupling of ester 1 with iodobenzene afforded a maximum yield of 74% of desired compound 2 with reticulated vitreous carbon (RVC) as the electrode material. Without the nickel catalyst, formation of compound 2 was completely suppressed and the Kolbe coupling dimer 3 was instead formed. Turning over to flow, RVC foam pieces were utilised as both graphite electrode plates, allowing for a larger surface area of both electrodes. At increased flow rates, the decarboxylative arylation was favoured over Kolbe dimerisation, enabling full conversion of 1 in a single pass; attributed to more efficient mixing. Final optimised conditions afforded a higher yield of 81% of compound 2 at a current density of 14 mA cm^−3^ and a residence time of 8.3 min. Shown below (Fig. 22), this electrochemical cell is commercially available and is called C-Flow LAB 1 × 1 (5 × 5 also available). The electrode area of the carbon used is 10 × 10 mm and had a Nafion membrane separating the electrodes. With a built-in stand, at 1200 g in weight, the unit is 110 mm high with a width and depth of 95 mm and 135 mm, respectively. The system has the added advantage of being able to use any electrode material, easy to assemble by hand and comes with templates for spacers and membranes.

## 3. Progress in electrochemical flow sono-microreactors

The major shortcomings of microreactors are inefficient reactant mixing and the clogging of microchannels due to solids forming/the occurrence of precipitation during reactions. In this perspective, efficient mass and energy transfers is a requirement. Numerous methods have been recommended to address this problem. The use of segmented liquid–liquid flow can prevent the particles from interacting with the reactor walls.^105–108^ Although this is an good way to deal with solids, the use of an additional solvent can reduce the efficiency of the reactions or be incompatible with the reaction mixture.^109^ Also, the presence of water can be unfavourable for the progress of many reactions. Sonochemistry could play a key role to overcome limitations caused by solid formations by introducing ultrasound in conventional flow systems^110^ and microreactors. We will pay attention to the latest developments and viewing future directions, which will open up the unique and unprecedented opportunity of sonoelectrochemistry of particulates. With the support of ultrasound, the mass transfer limitation in microreactors can now be partially overcome. On the other hand, the well-defined configuration of microreactors makes it easy to match with the ultrasonic field and provides an ideal platform to investigate and control the acoustic cavitation process.^110*b*^

**Fig. 23:**
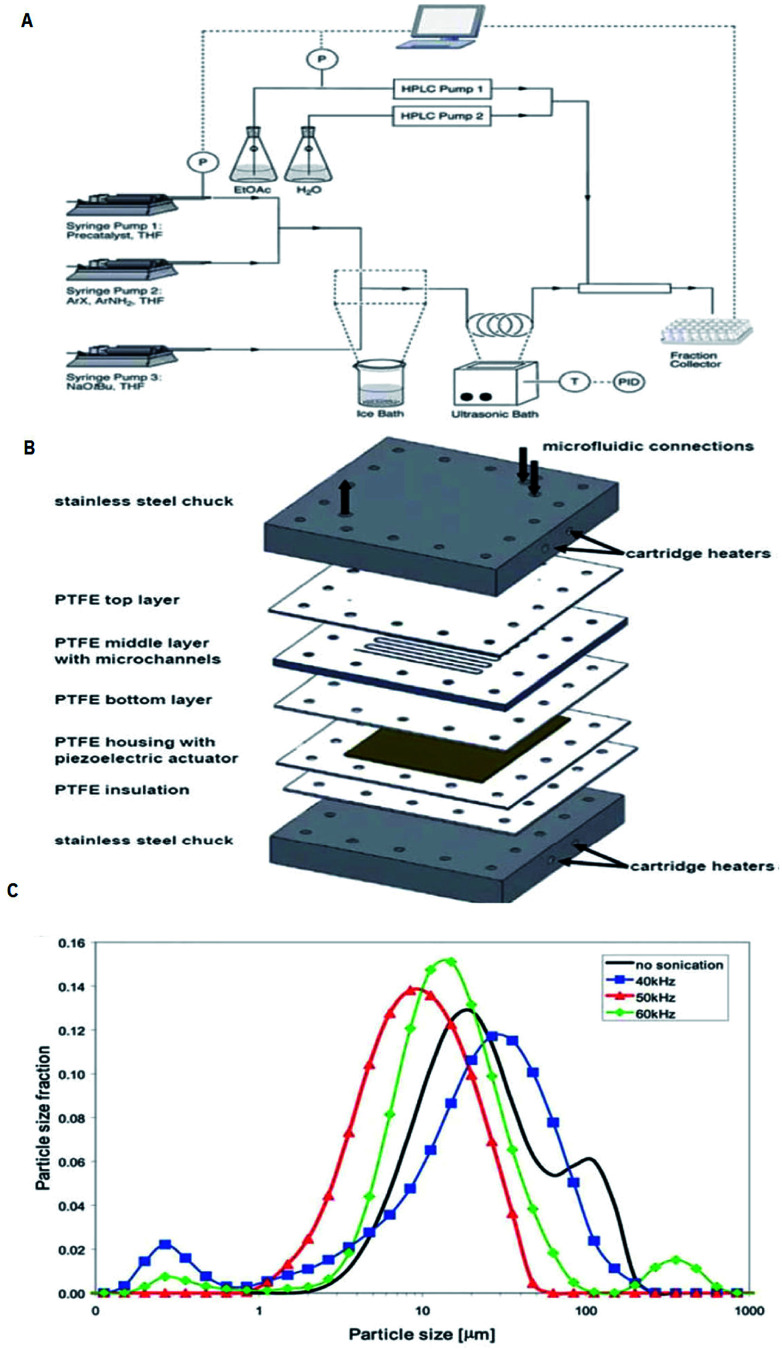
Examples of the use of ultrasound in microfluidic systems to avoid clogging. The methods of ultrasound integration range from immersing the reactor in an ultrasonic bath (a) to a full reactor assembly with the ultrasound transducer positioned next to the microfluidic channels (b). (A) Using an ultrasonic batch to handle solids formed during palladium-catalysed amination reactions, reproduced with permission from ref. 113, the Royal Society of Chemistry. (B) A Teflon microreactor with integrated piezoelectric actuator to handle solid forming reactions, reproduced from ref. 114, the Royal Society of Chemistry. The latter design eliminates the need of a transfer medium for the acoustic wave, and allows precise control of the applied ultrasound frequency and power. (C) The final precipitate particle size depends on the applied ultrasound frequency, and consequently a precise control of the operating parameters is desired for the combination of ultrasound and microfluidics, reproduced with permission from ref. 114, the Royal Society of Chemistry.

The incorporation of acoustic actuators with microstructures is a new and emerging area, where the acoustic energy is mostly supplied using transducers or piezoelectric microdevices with different sizes and geometries.^111,12*c*,112–115^ At increased power, acoustic irradiation has been revealed as successful in reducing agglomerate particle size, which is essential to prevent clogging.^12*c*,113,116^ A well-considered reaction system and also a challenge under flow settings due to clogging is that of Pd-catalysed bond formation reactions.^112^ Under typical reaction conditions inorganic by-products precipitate immediately in the apolar solvents needed for this conversion. One methodology to stop clogging is to immerse the Teflon tube containing the reaction mixture in an ultrasonic bath for irradiation, as shown in Fig. 23a.^113^ However, during the use of an ultrasonic bath, it should be noted that not one single frequency is excited, but the resulting waveform can create quite a complex outcome.^112^ Furthermore, the radiated ultrasonic waves initial need to pair with the media in the bath before transmitting to the microreactor. However, integrating a piezoelectric actuator directly into the microfluidic assembly to directly transmit the acoustic waveform to the reactor is energetically more effective. A model of such a multi-layered microreactor system is illustrated in Fig. 23b. This advanced home-made microsono-reactor has been developed by assembling PTFE plates (70 × 70 mm, channel width = 600 mm) with a piezoelectric actuator (1 mm thickness), the latter being driven at different frequencies by a wave generator and amplified to an optimal power of 30 W. This reactor was also used successfully in the Pd-catalysed cross-couplings and indorsed for long-term operation.^114^ The formation of gas bubbles upon ultrasonic irradiation lead to the breakup of the particle agglomerates.^117–119^ Moreover, using piezoelectric actuators allows for a precise control of the operating frequency, which is vital to control the resulting size of the agglomerates. Fig. 23c depicts the particle size distribution of inorganic precipitates subject to the applied ultrasound frequency, and for the particular setup the identified optimum frequency corresponded to 50 kHz. Sonication at 50 kHz prevented the microreactor from clogging (most particles were smaller than 20 mm), and excellent yields (>95%) could be obtained within 60–90 s. This suggests that solvent degassing and cavitation are largely responsible for mechanical effects for that particular experiment.

## 4. Summary and future directions

Extensive organic syntheses have been reported the use of flow microreactors. Their nature allows careful control of parameters (flow rate, reaction medium, current, reactant concentration) enabling the possibility to achieve products with higher purity and greater selectivity than their conventional batch counterparts. However, few examples are available on flow electrosynthesis through microreactors and rare examples of microsono-reactors. Microsono-reactors would potentially be useful to overcome the limitations to handle solid forming reactions. Various aforementioned flow methods for electrosynthesis through microreactors would definitely help to meet future demands for efficient synthesis and production of various pharmaceuticals and fine chemicals. Many efforts are needed to explore this new microreactor technology. The design of an electrochemical microreactor should enable usage in a simple and repeatable manner, have efficient heat and mass transfer and be as inexpensive as possible whilst allowing reactions to be performed efficiently and environmental friendly. The future potential for microreactor technology is exciting. Present-day limitations can be overcome, the ability to scale-up produce and open the doors for more convenient laboratory syntheses have clearly transformed this area into a hot topic. It will not be long before these cells are integrated as part of common practice amongst both academic and industrial laboratory processes. Flow is the next step in the evolution of chemistry and to continue this trend, microreactors must continue to impress in industrial productions. Further efforts will now be made by academics in an attempt to increase the use or microreactors as part of multistage flow syntheses and hence, the production of more complex compounds such as natural products and pharmaceuticals, which will in turn attract new financial backers. Scientists will also take this concept and apply it to areas of science and materials that lie outside the realm of organic chemistry.

## Conflicts of interest

There are no conflicts to declare.

## Supplementary Material
